# Assessing the risk of cataracts associated with medications: A pharmacovigilance analysis of the FAERS database

**DOI:** 10.1097/MD.0000000000049856

**Published:** 2026-07-17

**Authors:** Heng Chen, Ju Hou, Lihui Ouyang, Gefei He, Juanjuan Huang

**Affiliations:** a Department of Pharmacy, The First Hospital of Changsha (The Affiliated Changsha Hospital of Xiangya School of Medicine, Central South University), Changsha, Hunan, China.

**Keywords:** cataracts, disproportionality analysis, drug-induced risk, FAERS, pharmacovigilance

## Abstract

Cataracts are a leading cause of global blindness and visual impairment, with drug-induced cataracts emerging as a significant yet understudied contributor. This study aimed to comprehensively and systematically investigate medication-related cataract risk signals using data from the FDA Adverse Event Reporting System. We searched the FDA Adverse Event Reporting System database for all reported cases of medication-related cataracts from October 2014 to September 2024. Disproportionality analysis, employing the information component and reporting odds ratio, identified the medications most commonly associated with cataracts. Demographic characteristics, drug categories, and time-to-onset were also evaluated. After deduplication, 37,838 cataract-related reports were included. A total of 588 drugs were linked to cataracts. The top 50 medications with the highest signal association strength were identified based on information component values, including anti-cancer agents, immunomodulators, glucocorticoids, adrenergic medications, and anticholinergic medications. Mirvetuximab soravtansine, nitisinone, and belantamab mafodotin demonstrated the strongest signals. Most cases involved individuals over 60 years old, with females accounting for 68.7% of the reports. Significant variability in time-to-onset was also observed. In conclusion, this large-scale signal detection analysis utilizes real-world data to provide a comprehensive list of medications potentially associated with cataracts. Future research is required to validate these statistical associations.

## 1. Introduction

Cataracts are among the most widespread and debilitating ocular disorders, serving as a leading cause of both temporary and permanent visual impairment. Globally, they account for a significant proportion of blindness and vision loss. According to the Global Burden of Disease Study,^[1]^ approximately 15.2 million people are blind, and 78.8 million are visually impaired worldwide, with cataracts responsible for 45.4% of blindness and 38.9% of vision impairment cases. Nearly 15 million individuals aged over 50 have experienced vision loss attributable to cataracts. With the global population aging and life expectancy rising, it is projected that by 2025, around 40 million people will suffer from vision impairment due to cataracts.^[2]^ Cataract surgery is the standard intervention when the condition begins to significantly hinder an individual’s daily activities or quality of life. However, despite advances in surgical techniques, the prevalence of cataracts continues to increase,^[3]^ imposing substantial financial burdens on governments, communities, families, and individuals. Moreover, cataracts markedly diminish patients’ quality of life and adversely affect their mental health, underscoring the urgency of preventing and delaying their progression as a public health priority.

Understanding and addressing the risk factors associated with cataracts is essential. Aging, smoking, alcohol consumption, diabetes, metabolic syndrome, and obesity are well-established contributors to cataract development.^[2]^ In addition to these, certain medications play a significant role in cataractogenesis. For instance, glucocorticoids are strongly associated with the onset of posterior subcapsular cataracts.^[4,5]^ Clinically, these are characterized by centrally located posterior subcapsular opacities.^[6]^ Furthermore, several studies have demonstrated a strong positive correlation between the use of psychotropic medications and an increased risk of cataracts.^[7,8]^ The role of systemic medications, such as statins, has also been explored in cataractogenesis, although evidence remains inconclusive.^[9,10]^ Identifying drugs that contribute to cataract formation is critical for reducing the overall incidence of the disease. However, despite the known risks of specific drug classes, there is currently a lack of a comprehensive and systematic evaluation of medication-associated cataract signals utilizing large-scale real-world pharmacovigilance data.

Building on prior research into drug-induced ocular toxicities,^[11,12]^ this study is designed as an exploratory and hypothesis-generating analysis to investigate the relationship between various medications and cataracts by leveraging data from the FDA Adverse Event Reporting System (FAERS). Serving primarily as an exploratory and hypothesis-generating foundation, the objective is to uncover novel risk signals associated with drug-induced cataracts, utilizing large-scale, real-world evidence. The primary objective is to systematically screen and identify potential new risk signals associated with drug-induced cataracts through large-scale, real-world evidence. This research holds substantial clinical significance, offering insights to guide informed decision-making, optimize medication use, personalize treatment strategies, and ultimately mitigate the risk of drug-related cataracts.

## 2. Methods

### 2.1. Data source and collection

This study employs a real-world pharmacovigilance approach based on FAERS, a publicly accessible database that aggregates global spontaneous reports of adverse events (AEs). FAERS serves as a cornerstone of the US Food and Drug Administration’s (FDA) post-marketing surveillance efforts, offering valuable real-world insights into the occurrence of AEs. Reports are submitted voluntarily and are coded using preferred terms (PTs) from the Medical Dictionary for Regulatory Activities. The database is updated quarterly and is publicly accessible at https://fis.fda.gov/extensions/FPD-QDE-FAERS/FPD-QDE-FAERS.html.

FAERS comprises multiple data files, including DEMO (demographic and administrative data), DRUG (drug information), REAC (coded AEs), OUTC (patient outcomes), RPSR (reporting sources), THER (therapy initiation and termination dates), and INDI (drug indications). These datasets are interconnected within the database via unique identifiers such as the PRIMARYID. This retrospective analysis focused on data spanning from the fourth quarter of 2014 to the third quarter of 2024. As the study utilized publicly available and de-identified data, ethical approval was not required.

### 2.2. Data preprocessing and extraction

**Figure 1.**
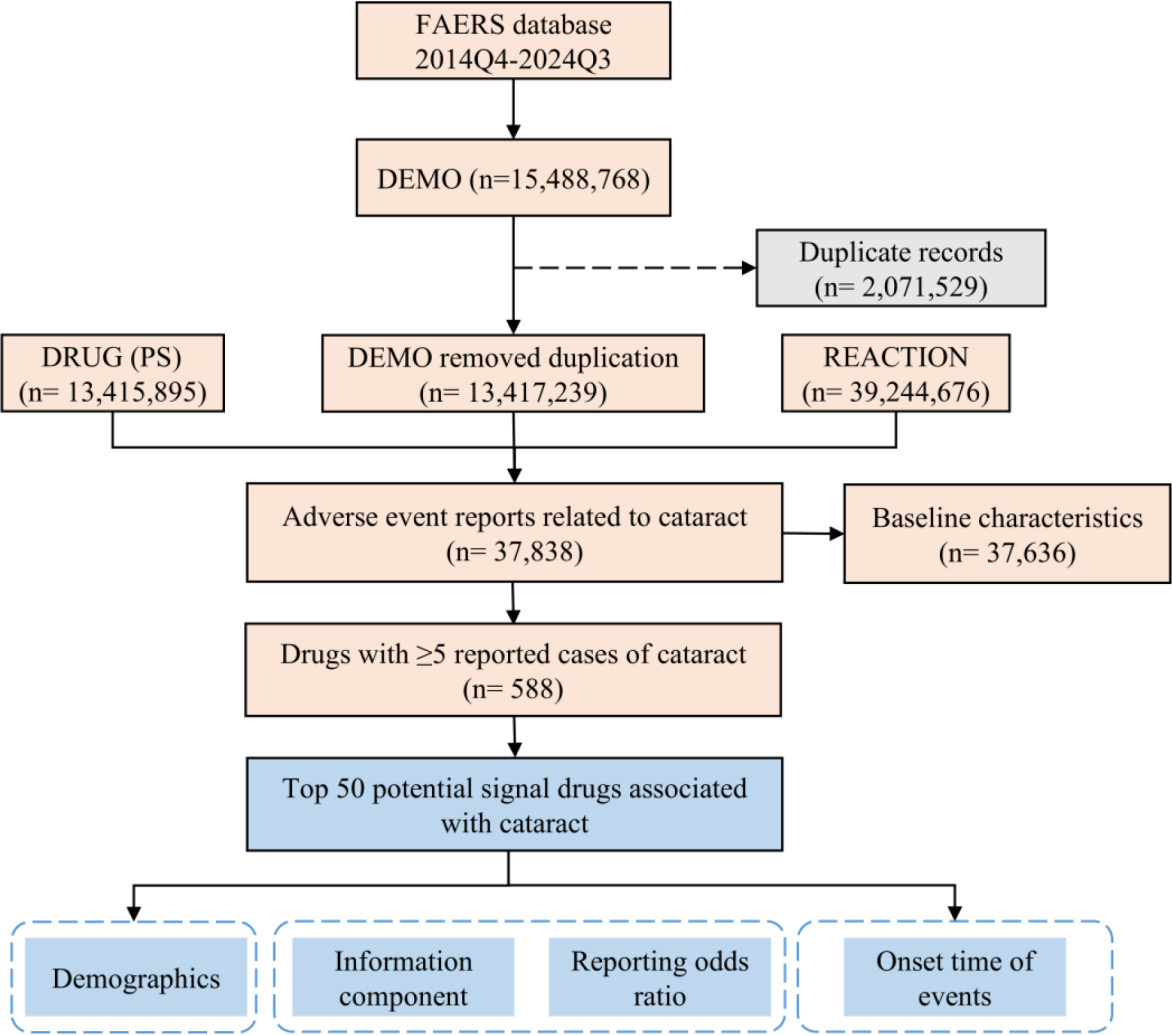
The flow chart of the study. DEMO = demographic and administrative data, DRUG = drug information, FAERS = FDA Adverse Event Reporting System.

A total of 15,488,768 reports were extracted and imported into SQLite 3.49.1 (SQLite Development Team) for analysis. In adherence to FDA guidelines, a variable-matching method was employed to resolve duplicate reports. Deduplication involved selecting the most recent and the highest PRIMARYID in instances where the CASEID was identical. This process resulted in the removal of 2,071,529 duplicate entries, as illustrated in Figure 1. Each AE report in FAERS is assigned a role code that specifies the suspected relationship between the drug and the event: “preferred suspect” (PS), “secondary suspect” (SS), “concomitant” (C), or “interacting” (I). For this study, only reports categorized under the role code “PS” were included to enhance analytical precision.^[13]^ Using the “Search by Reaction Term” method, we specifically queried for reports involving a single MedDRA PT, “Cataract” (MedDRA Code: 10007739). This focused, single-PT approach was intentionally chosen to ensure high diagnostic specificity and clarity in the retrieved cases, minimizing the inclusion of broader or ambiguous ocular conditions and thereby enhancing the reproducibility of our signal detection. Following deduplication and rigorous screening, a final dataset comprising 37,838 unique cataract-related AE reports was curated for further analysis.

### 2.3. Statistical analysis

Demographic variables, including patient gender, age, geographic region, and reported outcomes, were documented for all cataract-related AE reports. To identify potential drug-associated cataract risks, disproportionality analysis – a widely adopted method in pharmacovigilance for signal detection – was employed.^[14]^ Specifically, 2 well-established disproportionality metrics, the information component (IC) and the reporting odds ratio (ROR), were utilized to detect associations between drugs and cataract development. It is important to note that while these methods are effective for signal identification, they do not adjust for potential clinical confounders. It is critical to emphasize that while these disproportionality methods are highly effective for initial signal identification, they are inherently limited by their inability to adjust for potential clinical confounders, such as underlying comorbidities, concomitant medication use, and disease severity.

To bolster the robustness of the findings and mitigate false-negative signals, a statistical shrinkage transformation was applied to stabilize risk estimates, particularly when cell counts are small. The formulas used to compute the adjusted IC and ROR are detailed in Table 1. A signal was deemed statistically significant if the lower bound of the 95% confidence interval (CI) for IC (IC_025_) exceeded 0, or if the corresponding lower bound for ROR (ROR_025_) exceeded 1, provided there were at least 5 reported cases. Higher IC or ROR values generally indicate a stronger association with cataract risk. Furthermore, the study investigated the time to onset of cataracts associated with specific drugs, comparing onset durations across different drug classes identified as carrying positive signals for cataract risk. Time-to-onset analysis was restricted to 6130 reports (16.2% of total cases) that contained complete timing records (drug initiation and AE onset dates precise to the day).

**Table 1:** Major algorithms used for pharmacovigilance analysis.

Algorithms	Equation	Criteria
IC	$N_{expected}=\frac{\left(a+b\right)\left(a+c\right)}{a+b+c+d}$	IC_025_ > 0
	$IC=log_{2}\frac{a+0.5}{N_{expected}+0.5}$	
	$IC_{025}=IC-3\mathrm{.3}{\left(a+0.5\right)}^{-0.5}-2{\left(a+0\mathrm{.5}\right)}^{-1.5}$	
	$IC_{975}=IC+2.4{\left(a+0.5\right)}^{-0.5}-0.5{\left(a+0.5\right)}^{-1.5}$	
ROR	$ROR=\frac{a+0.5}{N_{expected}+0.5}$	ROR_025_ > 1
	$95\;\%\;CI=e^{ln\left(ROR\right)\pm 1.96\sqrt{\frac{1}{a}+\frac{1}{b}+\frac{1}{c}+\frac{1}{d}}}$	

95% CI = 95% confidence interval, a = number of reports containing both the target drug and target adverse event, b = number of reports containing other adverse events of the target drug, c = number of reports containing the target adverse event of other drugs, d = number of reports containing other drugs and other adverse events, IC = information component, ROR = reporting odds ratio.

All data extraction and preprocessing steps were independently conducted by 2 authors to ensure reliability. Data processing and deduplication were performed using SQLite 3.49.1, while statistical analyses were executed using Python 3.10 (Python Software Foundation) and SPSS 27.0 (IBM Corp).

### 2.4. Ethics approval

Because FAERS is a publicly available and anonymized database, this study did not require ethical approval.

## 3. Results

### 3.1. Characteristics of subjects

**Figure 2.**
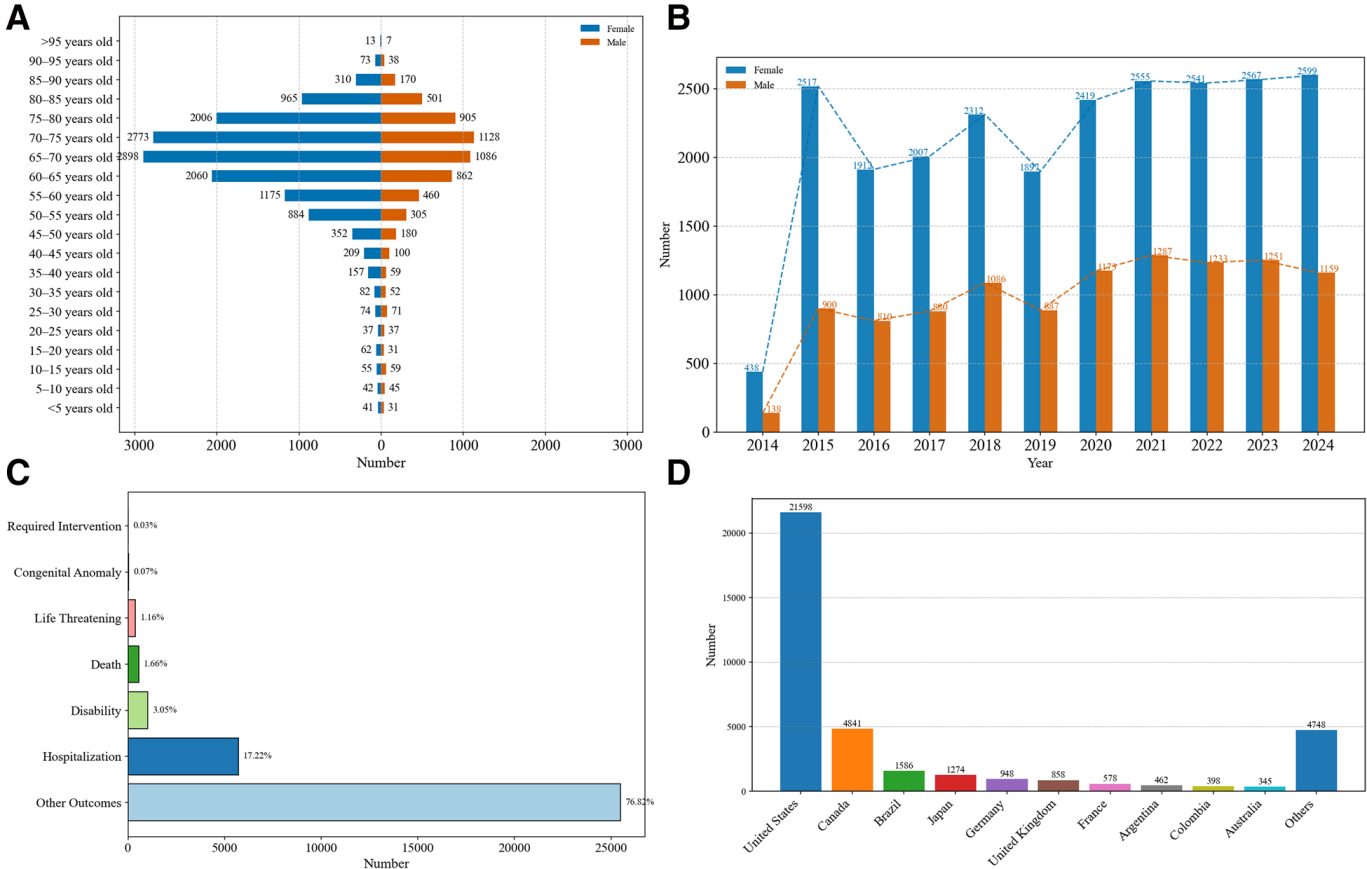
Demographic distribution of drug-related cataracts. (A) Age and gender-based population pyramid of individuals with drug-related cataracts. (B) Annual reporting trends of drug-related cataract cases stratified by gender. (C) Outcome distribution across affected individuals. (D) Geographic distribution of reporting countries for cases.

An analysis of the FAERS database from Q4 2014 to Q3 2024 identified 37,636 subjects with cataracts. The mean age of the affected individuals was 65.6 ± 26.9 years, with females constituting the majority (68.7%). The age distribution of drug-induced cataracts was predominantly concentrated in individuals aged 60 to 80 years, regardless of gender (Fig. 2A). Over the past decade, the annual number of reported cases has remained relatively stable, although the incidence consistently exhibited a higher prevalence in females compared with males (Fig. 2B). The most frequently reported clinical outcomes were classified as “other serious outcomes (important medical event)” (76.8%) and “hospitalization (initial or prolonged)” (17.2%; Fig. 2C). Notably, this high proportion of serious outcomes may reflect the inherent reporting bias of the FAERS database, which tends to overrepresent medically significant events. Geographically, the majority of reports originated from the United States (57.4%) and Canada (12.9%; Fig. 2D).

### 3.2. Distribution of drug categories associated with cataracts

**Figure 3.**
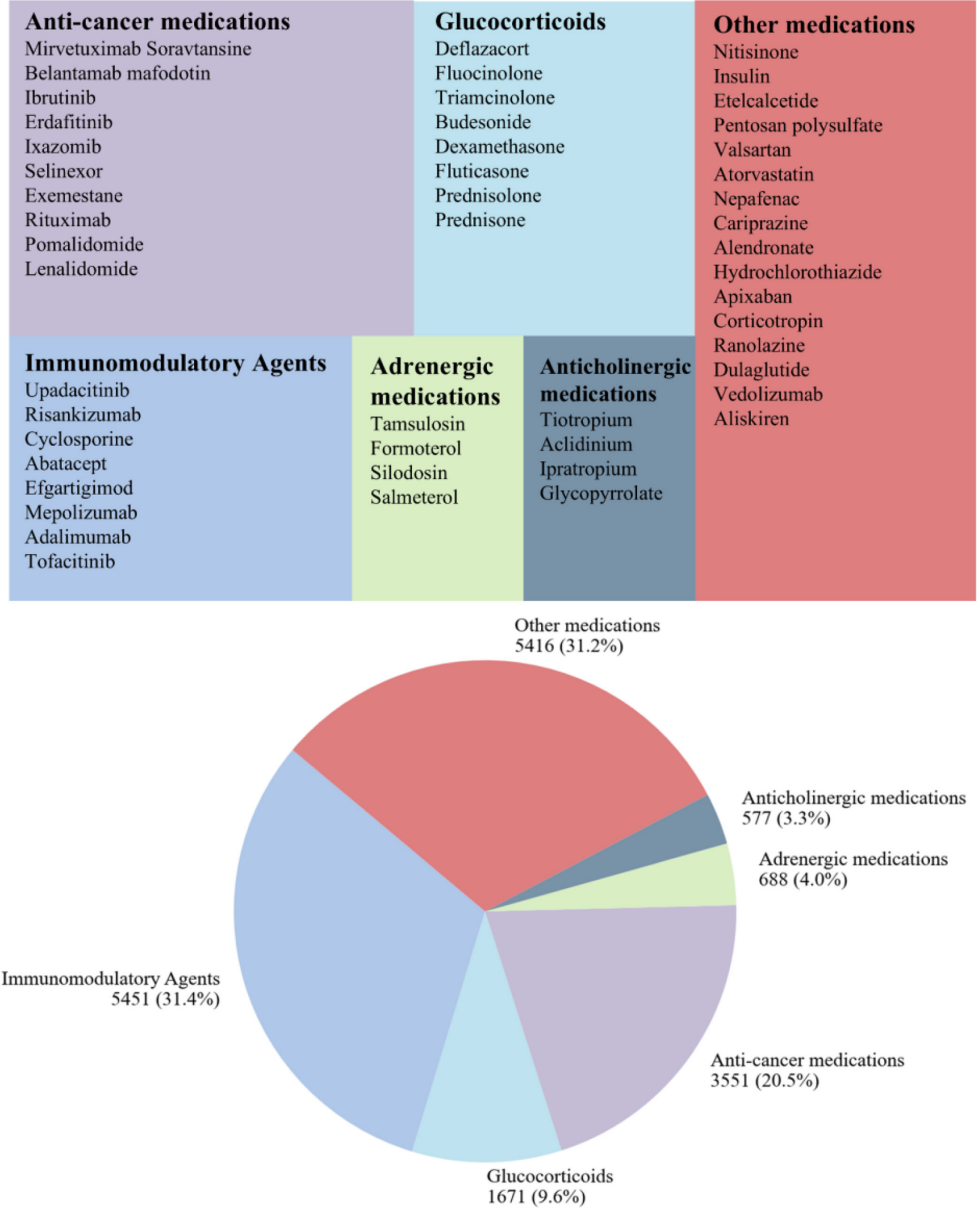
Categorization of drugs inducing cataracts based on their underlying mechanisms of action.

A disproportionality analysis was performed on 588 drugs implicated in cataract-related AEs. After excluding drugs explicitly used to treat ocular conditions, the top 50 drugs were ranked based on the strength of their signal. Among these, the distribution was as follows: 10 anti-cancer drugs (20%), 8 glucocorticoids (16%), 8 immunomodulatory agents (16%), 4 adrenergic medications (8%), and 4 anticholinergic medications (8%) (Fig. 3). Notably, immunomodulatory agents accounted for the highest number of individual reports, with 5451 cases.

### 3.3. Risk assessment of drugs associated with cataracts

**Figure 4.**
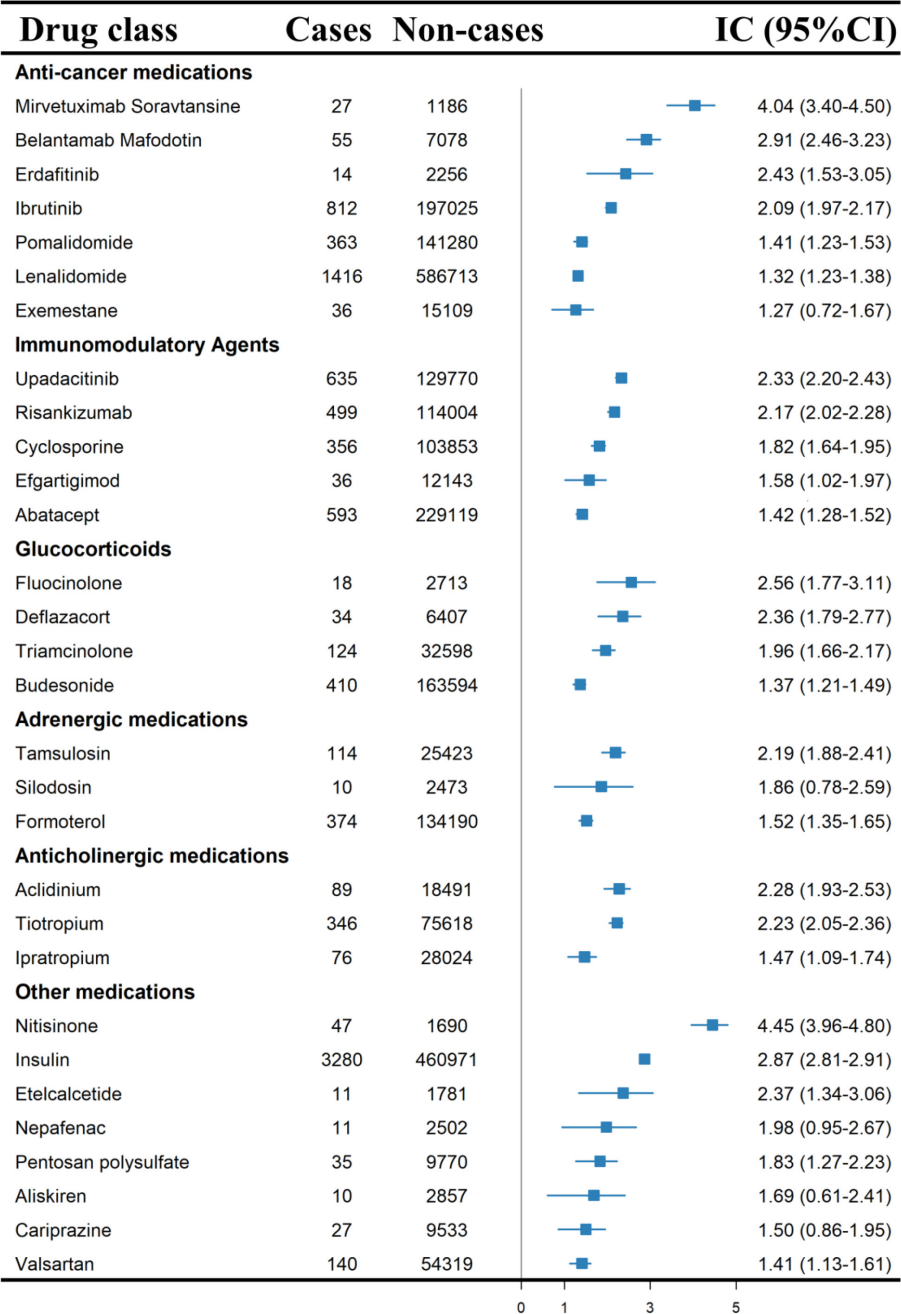
The top 30 potential signal drugs increasing cataract risk. IC = information component.

To improve readability and prioritize clinically relevant findings over an exhaustive listing, we highlight only the most significant signals (top 30) across key drug categories (Fig. 4). Within the anti-cancer category, antibody-drug conjugates such as mirvetuximab soravtansine (IC: 4.04, 95% CI: 3.40–4.50) and belantamab mafodotin (IC: 2.91, 95% CI: 2.46–3.23) demonstrated the highest signal strengths. For immunomodulatory agents, upadacitinib (IC: 2.33, 95% CI: 2.20–2.43) and risankizumab (IC: 2.17, 95% CI: 2.02–2.28) exhibited prominent associations. In the glucocorticoid class, fluocinolone (IC: 2.56, 95% CI: 1.77–3.11) remained the top-ranked medication. Among urological and respiratory treatments, tamsulosin (IC: 2.19, 95% CI: 1.88–2.41) and aclidinium (IC: 2.28, 95% CI: 1.93–2.53) showed notable signals. In addition, among other medications, nitisinone (IC: 4.45, 95% CI: 3.96–4.80) and insulin (IC: 2.87, 95% CI: 2.81–2.91) presented exceptionally strong associations. These associations should be interpreted with caution as it likely reflects confounding by indications. A categorical grouping of 6 drug classes was conducted, with subsequent disproportionality analyses revealing that both IC and ROR values consistently indicated significant risks of cataract development across these categories (Table 2).

**Table 2:** Disproportionality analysis of reports involving 6 major classes of drugs associated with cataracts.

Drug	Cases	Non-cases	IC (95% CI)	ROR (95% CI)
Anti-cancer medications	3551	1,414,336	1.38 (1.32–1.42)	2.60 (2.51–2.69)
Immunomodulatory agents	5451	2,425,291	1.22 (1.17–1.25)	2.33 (2.26-2.39)
Glucocorticoids	1671	755,651	1.19 (1.11–1.25)	2.29 (2.18–2.40)
Adrenergic medications	688	264,674	1.43 (1.30–1.52)	2.69 (2.49–2.90)
Anticholinergic medications	577	153,696	1.95 (1.81–2.05)	3.87 (3.56–4.20)
Other medications	5416	1,509,163	1.89 (1.85–1.92)	3.71 (3.60–3.82)

CI = confidence interval, IC = information component, ROR = reporting odds ratio.

### 3.4. Drug-induced time analysis

**Figure 5.**
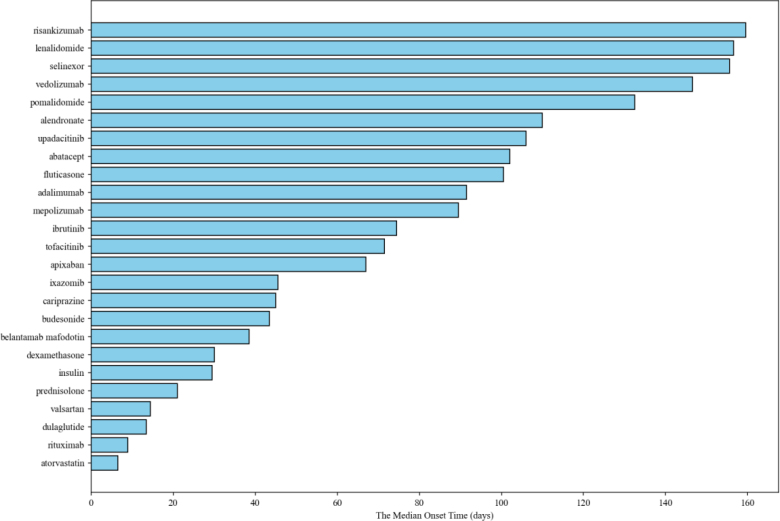
Time to onset of drug-related cataracts.

Due to the incomplete documentation of the timing of drug administration and the onset of AEs, only drugs with at least 10 reports containing complete time data were included in the analysis of median onset times. As depicted in Figure 5, significant variability in median onset times was observed among different drugs. Risankizumab had the longest median onset time at 159.5 days, whereas cyclosporine exhibited the shortest median onset time at 6.5 days.

## 4. Discussion

This study presents a comprehensive analysis of pharmaceutical agents potentially linked to an elevated risk of cataracts over the past decade. Utilizing IC values, we identified the top 50 drugs exhibiting the strongest risk signals, encompassing diverse categories such as anti-cancer medications, immunomodulators, glucocorticoids, adrenergic medications, and anticholinergic medications. Furthermore, we examined the variability in the onset time of drug-induced cataracts. To our knowledge, this investigation represents a large-scale evaluation leveraging real-world evidence from the FAERS database to explore the statistical associations between FDA-approved drugs and cataract risk. The findings not only provide pivotal real-world data to enhance the assessment of ocular toxicity signals but also support strategies to mitigate potential drug-induced cataract risk.

Our analysis revealed a higher frequency of cataract cases among women, corroborating prior studies.^[15]^ Gender appears to play a pivotal role in disease susceptibility, progression, and therapeutic outcomes.^[16]^ The elevated cataract risk observed in women has been hypothesized to be attributable to the involvement of estrogen in cataractogenesis. Estrogen’s antioxidant properties are thought to counteract reactive oxygen species, a primary driver of cataract formation. However, postmenopausal hormonal changes may diminish this protective effect, potentially heightening susceptibility to cataracts.^[17]^ In addition, socioeconomic inequities – such as disparities in education, employment, income, and healthcare access – may exacerbate this risk among women.^[18]^ These findings underscore the need for targeted research to elucidate the mechanisms linking gender and drug-induced cataracts. Cataract cases predominantly occurred in individuals aged 60 years and older. This is widely hypothesized to be primarily attributed to aging, which is thought to lead to increased oxidative stress and protein accumulation in the lens, potentially impairing its transparency and thereby elevating the risk of cataracts.^[19]^ As such, elderly patients prescribed high-risk medications require vigilant monitoring for ocular AEs. Notably, our analysis also identified significant variability in the latency periods of cataract-related AEs across different drug classes, with shorter latency periods warranting particular caution in clinical practice to enable timely detection and intervention.

Anti-cancer agents emerged as a prominent category associated with ocular toxicities, including conjunctivitis and keratitis.^[20]^ Within this group, antibody-drug conjugates such as mirvetuximab soravtansine and belantamab mafodotin demonstrated the most pronounced cataract risk signals. Mirvetuximab soravtansine, previously linked to ocular AEs like dry eye, blurred vision, and keratopathy in clinical trials,^[21,22]^ exhibited a robust correlation with cataracts in our analysis. Similarly, belantamab mafodotin, which caused ocular toxicities in approximately 70% of patients in the DREAMM-2 trial, primarily manifesting as corneal epithelial changes,^[23]^ was strongly associated with cataract risk signals in our study. Exposure-safety analyses revealed that higher drug concentrations increase the likelihood of grade 2 or 3 corneal AEs. Animal studies have further suggested that belantamab mafodotin can penetrate ocular tissues via tear-associated vascular pathways.^[24]^ These findings highlight the need for further investigations to delineate the underlying mechanisms, which are hypothesized to involve both target-mediated effects and off-target toxicities.^[11]^

Tanaka et al conducted an analysis based on the Japanese pharmacovigilance database and similarly identified a strong risk association between lenalidomide/pomalidomide and lens disorders.^[25]^ In a phase II study on primary vitreoretinal lymphoma, 54.45% of patients treated with lenalidomide and rituximab experienced grade 3 cataracts.^[26]^ The small-molecule inhibitor ibrutinib, a Bruton’s tyrosine kinase inhibitor, has been previously associated with adverse ocular events such as red eye or dry eye syndrome.^[20]^ Our research further suggests that ibrutinib may also carry a potential risk for cataracts. For erdafitinib, a tyrosine kinase inhibitor targeting fibroblast growth factor receptors, multiple case reports have documented its lens toxicity, contributing to the onset of cataracts.^[27,28]^ In a long-term follow-up of a phase II clinical trial of erdafitinib, 2 out of 101 patients developed cataracts after a median treatment exposure of 5.4 months.^[29]^ Fibroblast growth factor receptor signaling has been implicated in various aspects of lens development, including lens cell proliferation and overall survival.^[30]^ Further investigations are warranted to elucidate the precise mechanisms by which these antineoplastic agents may potentially induce cataract formation.

Glucocorticoids are well documented to induce posterior subcapsular cataracts,^[31]^ with oral formulations posing a higher risk than inhaled ones.^[4]^ Our findings corroborate this, showing significant associations between various glucocorticoids and cataracts, with inhaled agents like budesonide and fluticasone exhibiting weaker links than oral glucocorticoids. The underlying mechanisms are hypothesized to involve glucocorticoid receptor activation in lens epithelial cells and the formation of glucocorticoid-protein adducts.^[6,32,33]^ In addition, β2-adrenergic receptor agonists (e.g., formoterol and salmeterol) and anticholinergic medications, frequently co-administered with glucocorticoids for respiratory diseases such as chronic obstructive pulmonary disease and asthma, may influence cataract risk.^[34]^ Furthermore, corticotropin, which stimulates endogenous glucocorticoid secretion, also demonstrated a notable risk signal in our study.

Adrenergic and anticholinergic agents have been implicated in drug-induced glaucoma through hypothesized mechanisms such as pupillary block.^[35]^ Certain cholinergic drugs may induce glaucoma by causing anterior displacement of the lens-iris diaphragm.^[36]^ While selective α1-adrenergic receptor antagonists have not been directly linked to cataract formation, they have been associated with an elevated risk of intraoperative complications during cataract surgery.^[37]^ In our study, both tamsulosin and silodosin showed associations with reported cataracts.

Immunomodulatory agents, widely used for autoimmune diseases, also show potential cataract risk signals. Upadacitinib and tofacitinib, which specifically target the JAK-STAT signaling pathway, have been hypothesized to contribute to cataractogenesis through mechanisms such as lens epithelial cell proliferation potentially induced by JAK/STAT signaling dysregulation.^[38]^ Wu et al also reported an association between upadacitinib and cataracts.^[39]^ Similarly, the interleukin-23 inhibitor risankizumab has been linked to cataracts,^[40]^ although this AE is not currently listed in its product label. For cyclosporine and adalimumab, commonly used in uveitis treatment, the risk of cataract may be confounded by the underlying disease itself, as uveitis frequently leads to cataracts as a complication.^[41]^ This suggests that the risk signal may reflect the severity of the inflammatory condition rather than a direct drug effect. Observational studies suggest that autoimmune diseases may independently increase cataract risk.^[42,43]^ This increased susceptibility may be due to oxidative stress in autoimmune patients, which leads to elevated oxidative pressure in the lens, reduced reactive oxygen species-scavenging capacity, and lipid peroxidation, ultimately predisposing these individuals to cataract formation.^[44]^ Further research is required to delineate the causal relationship between immunomodulatory agents and cataract development.

Among other drug classes, nitisinone, a rare disease drug used for hereditary tyrosine metabolism disorders, exhibited a strong risk signal. A long-term follow-up study over 5 years reported a significant increase in lens opacity among patients treated with nitisinone.^[45]^ Insulin also demonstrated a robust association with cataracts and accounted for the highest number of reported cases. The Beaver Dam Eye Study identified a significant increase in the 5-year cataract incidence rate among insulin users.^[46]^ Interestingly, aside from dulaglutide, other antidiabetic medications showed no strong association with cataract risk. This finding aligns with Deng et al’s analysis of National Health and Nutrition Examination Survey data, which demonstrated that after adjusting for confounding factors, only insulin use remained significantly associated with cataracts.^[47]^ The persistent link between insulin and cataracts may stem from hypothesized mechanisms such as an unfolded protein response and apoptosis of lens epithelial cells or from more severe or poorly controlled diabetes in insulin users.^[48]^ For other medications, including diuretics,^[46]^ statins,^[9]^ and nonsteroidal anti-inflammatory drugs,^[46,49]^ the associations with cataracts remain controversial. However, our study identified significant risk signals for hydrochlorothiazide, atorvastatin, and nepafenac. Given the limitations of spontaneous reporting data, these results should be interpreted as associations requiring further validation through prospective clinical studies to clarify potential causal mechanisms.

Despite the valuable insights provided by this study, several limitations must be acknowledged. First, as FAERS is a spontaneous reporting system, it is inherently subject to reporting bias and underreporting, and the lack of denominator data (the total number of patients exposed to the medications) precludes the calculation of true incidence rates or absolute risk. Second, the potential risk of false-positive signals due to multiple testing should be considered, as conducting numerous statistical comparisons across a wide range of drugs may increase the likelihood of finding significant associations by chance. Third, the “Weber effect” – a phenomenon where reporting rates peak during the early post-marketing period due to stimulated reporting – may have influenced the signal strength for recently approved medications. Furthermore, this study is subject to residual confounding, as the FAERS database often lacks detailed clinical information regarding patients’ underlying comorbidities or disease severity, which could independently influence the risk of cataract development. Finally, the absence of precise data on drug dosage and the exact duration of exposure limits the interpretation of our findings, particularly regarding dose–response relationships or the cumulative effect of medication use. Consequently, while these signals provide important safety leads, they should be further validated through prospective clinical trials or robust longitudinal epidemiological studies.

## 5. Conclusion

This study represents a comprehensive pharmacovigilance analysis of drug-induced cataracts based on disproportionality analysis. This study identified the demographic and epidemiological characteristics of potential drug-induced cataracts and provided a potential list of pharmacovigilance signals for medications commonly associated with cataracts, including anti-cancer agents, immunomodulators, glucocorticoids, adrenergic medications, and anticholinergic medications. While these findings represent signals rather than confirmed drug-induced cataract risks, our list may contribute to better clinical management of the risk of drug-induced cataract events. Future research should focus on elucidating the biological mechanisms underpinning these associations and validating the findings through prospective studies.

## Author contributions

**Conceptualization:** Heng Chen, Ju Hou, Lihui Ouyang, Gefei He, Juanjuan Huang.

**Data curation:** Heng Chen, Lihui Ouyang, Juanjuan Huang.

**Formal analysis:** Heng Chen, Juanjuan Huang.

**Funding acquisition:** Heng Chen, Ju Hou.

**Investigation:** Heng Chen, Ju Hou.

**Methodology:** Heng Chen, Juanjuan Huang.

**Software:** Heng Chen.

**Supervision:** Heng Chen, Ju Hou, Lihui Ouyang, Juanjuan Huang.

**Visualization:** Heng Chen.

**Project administration:** Lihui Ouyang, Gefei He.

**Resources:** Gefei He.

**Validation:** Gefei He.

**Writing – original draft:** Heng Chen, Juanjuan Huang.

**Writing – review & editing:** Heng Chen, Ju Hou, Lihui Ouyang, Gefei He, Juanjuan Huang.

## References

[1] Blindness GBD, Vision Impairment C, Vision Loss Expert Group of the Global Burden of Disease S. Causes of blindness and vision impairment in 2020 and trends over 30 years, and prevalence of avoidable blindness in relation to VISION 2020: the Right to Sight: an analysis for the Global Burden of Disease Study. Lancet Glob Health. 2021;9:e144–e60.33275949

[2] Liu YC Wilkins M Kim T Malyugin B Mehta JS. Cataracts. Lancet (London, England). 2017;390:600–12. 28242111 10.1016/S0140-6736(17)30544-5

[3] Flaxman SR Bourne RRA Resnikoff S. Global causes of blindness and distance vision impairment 1990-2020: a systematic review and meta-analysis. Lancet Glob Health. 2017;5:e1221–34. 29032195 10.1016/S2214-109X(17)30393-5

[4] Wang JJ Rochtchina E Tan AG Cumming RG Leeder SR Mitchell P. Use of inhaled and oral corticosteroids and the long-term risk of cataract. Ophthalmology. 2009;116:652–7. 19243828 10.1016/j.ophtha.2008.12.001

[5] Carlson J McBride K O’Connor M. Drugs associated with cataract formation represent an unmet need in cataract research. Front Med (Lausanne). 2022;9:947659. 36045926 10.3389/fmed.2022.947659 PMC9420850

[6] James ER. The etiology of steroid cataract. J Ocul Pharmacol Ther. 2007;23:403–20. 17900234 10.1089/jop.2006.0067

[7] Erie JC Brue SM Chamberlain AM Hodge DO. Selective serotonin reuptake inhibitor use and increased risk of cataract surgery: a population-based, case-control study. Am J Ophthalmol. 2014;158:192–7.e1. 24631758 10.1016/j.ajo.2014.03.006 PMC4356987

[8] Chou PH Chu CS Chen YH. Antidepressants and risk of cataract development: a population-based, nested case-control study. J Affect Disord. 2017;215:237–44. 28342338 10.1016/j.jad.2017.03.044

[9] Yu S Chu Y Li G Ren L Zhang Q Wu L. Statin use and the risk of cataracts: a systematic review and meta-analysis. J Am Heart Assoc. 2017;6:e004180. 28320745 10.1161/JAHA.116.004180 PMC5523994

[10] Alves C Mendes D Marques FB. Statins and risk of cataracts: a systematic review and meta-analysis of observational studies. Cardiovasc Ther. 2018;36:e12480. 30597753 10.1111/1755-5922.12480

[11] Chen H Yang G Ma J. Ocular toxicity associated with anti-HER2 agents in breast cancer: a pharmacovigilance analysis using the FAERS database. Int J Cancer. 2024;154:1616–25. 38216995 10.1002/ijc.34848

[12] Ma J Chen W Hu Z. Rare ocular toxicity induced by pertuzumab/QL1209 in healthy Chinese subjects: case reports and whole-exome sequencing analysis. Invest New Drugs. 2022;40:861–7. 35596833 10.1007/s10637-022-01256-0

[13] Zhou Y Xie W Wang L. Anti-tumor necrosis factor-alpha therapy and hypoglycemia: a real-world pharmacovigilance analysis. Drug Saf. 2022;45:951–9. 35857191 10.1007/s40264-022-01210-2

[14] Almenoff JS Pattishall EN Gibbs TG DuMouchel W Evans SJ Yuen N. Novel statistical tools for monitoring the safety of marketed drugs. Clin Pharmacol Ther. 2007;82:157–66. 17538548 10.1038/sj.clpt.6100258

[15] Jiang B Wu T Liu W Liu G Lu P. Changing trends in the global burden of cataract over the past 30 years: retrospective data analysis of the global burden of disease study 2019. JMIR Public Health Surveill. 2023;9:e47349. 38051579 10.2196/47349 PMC10731550

[16] Mauvais-Jarvis F Merz NB Barnes PJ. Sex and gender: modifiers of health, disease, and medicine. Lancet (London, England). 2020;396:565–82. 32828189 10.1016/S0140-6736(20)31561-0 PMC7440877

[17] Zetterberg M Celojevic D. Gender and cataract – the role of estrogen. Curr Eye Res. 2015;40:176–90. 24987869 10.3109/02713683.2014.898774

[18] Courtright P Lewallen S. Why are we addressing gender issues in vision loss? Community Eye Health. 2009;22:17–9. 19888362 PMC2760274

[19] You L Lin Y Zheng Y Han Z Zeng L Chen H. The impact of aging on ocular diseases: unveiling complex interactions. Aging Dis. 2024;16:2803–30. 39500360 10.14336/AD.2024.0850 PMC12339180

[20] Kunkler AL Binkley EM Mantopoulos D. Known and novel ocular toxicities of biologics, targeted agents, and traditional chemotherapeutics. Graefes Arch Clin Exp Ophthalmol. 2019;257:1771–81. 31098752 10.1007/s00417-019-04337-8

[21] Moore KN Angelergues A Konecny GE. Mirvetuximab soravtansine in FRα-positive, platinum-resistant ovarian cancer. N Engl J Med. 2023;389:2162–74. 38055253 10.1056/NEJMoa2309169

[22] Secord AA Lewin SN Murphy CG. The efficacy and safety of mirvetuximab soravtansine in FRα-positive, third-line and later, recurrent platinum-sensitive ovarian cancer: the single-arm phase 2 PICCOLO trial. Ann Oncol. 2025;36:321–30. 39617145 10.1016/j.annonc.2024.11.011

[23] Lonial S Lee HC Badros A. Belantamab mafodotin for relapsed or refractory multiple myeloma (DREAMM-2): a two-arm, randomised, open-label, phase 2 study. Lancet Oncol. 2020;21:207–21. 31859245 10.1016/S1470-2045(19)30788-0

[24] Farooq AV Esposti SD Popat R. Corneal epithelial findings in patients with multiple myeloma treated with antibody-drug conjugate belantamab mafodotin in the pivotal, randomized, DREAMM-2 study. Ophthalmol Ther. 2020;9:889–911. 32712806 10.1007/s40123-020-00280-8 PMC7708586

[25] Tanaka J Koseki T Kondo M Ito Y Yamada S. Analyses of ocular adverse reactions associated with anticancer drugs based on the Japanese pharmacovigilance database. Anticancer Res. 2022;42:4439–51. 36039456 10.21873/anticanres.15944

[26] Zhang Y Zhang X Zou D. Lenalidomide and rituximab regimen combined with intravitreal methotrexate followed by lenalidomide maintenance for primary vitreoretinal lymphoma: a prospective phase II study. Front Oncol. 2021;11:701507. 34249763 10.3389/fonc.2021.701507 PMC8264769

[27] Bauters G Paques M Borderie V Bouheraoua N. Reversible corneal stromal thinning, acute-onset white cataract and angle-closure glaucoma due to erdafitinib, a fibroblast growth factor receptor inhibitor: report of three cases. J Fr Ophtalmol. 2021;44:67–75. 33162180 10.1016/j.jfo.2020.03.018

[28] Sassine AG Cakir Y Vecchia LD Ehlers JP. Erdafitinib-associated retinal alterations and rapid onset bilateral white cataracts. Am J Ophthalmol Case Rep. 2024;34:102028. 38572298 10.1016/j.ajoc.2024.102028 PMC10987836

[29] Siefker-Radtke AO Necchi A Park SH. Efficacy and safety of erdafitinib in patients with locally advanced or metastatic urothelial carcinoma: long-term follow-up of a phase 2 study. Lancet Oncol. 2022;23:248–58. 35030333 10.1016/S1470-2045(21)00660-4

[30] Lovicu FJ McAvoy JW de Iongh RU. Understanding the role of growth factors in embryonic development: insights from the lens. Philos Trans R Soc Lond B Biol Sci. 2011;366:1204–18. 21402581 10.1098/rstb.2010.0339 PMC3061110

[31] Smeeth L Boulis M Hubbard R Fletcher AE. A population based case-control study of cataract and inhaled corticosteroids. Br J Ophthalmol. 2003;87:1247–51. 14507760 10.1136/bjo.87.10.1247 PMC1920769

[32] James ER Fresco VM Robertson LL. Glucocorticoid-induced changes in the global gene expression of lens epithelial cells. J Ocul Pharmacol Ther. 2005;21:11–27. 15718824 10.1089/jop.2005.21.11

[33] James ER Robertson L Ehlert E Fitzgerald P Droin N Green DR. Presence of a transcriptionally active glucocorticoid receptor alpha in lens epithelial cells. Invest Ophthalmol Vis Sci. 2003;44:5269–76. 14638726 10.1167/iovs.03-0401

[34] Aydin Guclu O Ismayilov AS. Ocular surface, intraocular pressure and lens condition in bronchodilator and steroid-treated patients with chronic pulmonary disease. Med Princ Pract. 2023;32:288–96. 37725938 10.1159/000534172 PMC10659703

[35] Wu SN Chen XD Yan D. Drug-associated glaucoma: a real-world study based on the Food and Drug Administration adverse event reporting system database. Clin Exp Ophthalmol. 2025;53:140–60. 39460378 10.1111/ceo.14454

[36] Wicinski M Kaluzny BJ Liberski S Marczak D Seredyka-Burduk M Pawlak-Osinska K. Association between serotonin-norepinephrine reuptake inhibitors and acute angle closure: what is known? Surv Ophthalmol. 2019;64:185–94. 30278181 10.1016/j.survophthal.2018.09.006

[37] Wang YH Huang LC Tsai SHL Chen YJ Wu CL Kang YN. Risk of intraoperative floppy iris syndrome among selective alpha-1 blockers – a consistency model of 6,488 cases. Front Med (Lausanne). 2022;9:941130. 36111121 10.3389/fmed.2022.941130 PMC9468244

[38] Zhao Y Liu S Li X. Cross-talk of signaling pathways in the pathogenesis of allergic asthma and cataract. Protein Pept Lett. 2020;27:810–22. 32031062 10.2174/0929866527666200207113439

[39] Wu XP Lu XK Wang ZT. Post-marketing safety concerns with upadacitinib: a disproportionality analysis of the FDA adverse event reporting system. Expert Opin Drug Saf. 2023;22:975–84. 37310063 10.1080/14740338.2023.2223952

[40] Shu Y Chen J Ding Y Zhang Q. Adverse events with risankizumab in the real world: postmarketing pharmacovigilance assessment of the FDA adverse event reporting system. Front Immunol. 2023;14:1169735. 37256136 10.3389/fimmu.2023.1169735 PMC10225532

[41] Sada I Hiyama T Orihashi Y. Early immunosuppressive therapy and ocular complications in pediatric and young adult patients with non-infectious uveitis at a tertiary referral center in Japan. Ocul Immunol Inflamm. 2024;32:2459–66. 39436950 10.1080/09273948.2024.2409394

[42] Alderaan K Sekicki V Magder LS Petri M. Risk factors for cataracts in systemic lupus erythematosus (SLE). Rheumatol Int. 2015;35:701–8. 25257763 10.1007/s00296-014-3129-5

[43] Cheng CY. Risk of incident cataract in patients with psoriasis: a population-based cohort study. J Dermatol. 2022;49:359–67. 34862667 10.1111/1346-8138.16261

[44] Yuan W Li X Wang G Qu B Zhao F. Association of autoimmune and allergic diseases with senile cataract: a bidirectional two-sample Mendelian randomization study. Front Immunol. 2024;15:1325868. 38585265 10.3389/fimmu.2024.1325868 PMC10995295

[45] Ahmad MSZ Ahmed M Khedr M. Association of alkaptonuria and low dose nitisinone therapy with cataract formation in a large cohort of patients. JIMD Rep. 2022;63:351–60. 35822094 10.1002/jmd2.12288 PMC9259401

[46] Klein BE Klein R Lee KE Danforth LG. Drug use and five-year incidence of age-related cataracts: the Beaver Dam Eye Study. Ophthalmology. 2001;108:1670–4. 11535471 10.1016/s0161-6420(01)00656-x

[47] Deng R Zhu Z Han X. Evaluation of systemic medications associated with surgically treated cataract among US adults. Am J Ophthalmol. 2023;249:126–36. 36646239 10.1016/j.ajo.2023.01.005

[48] Becker C Schneider C Aballea S. Cataract in patients with diabetes mellitus-incidence rates in the UK and risk factors. Eye (Lond). 2018;32:1028–35. 29386666 10.1038/s41433-017-0003-1 PMC5997651

[49] Chang MA Congdon NG Bykhovskaya I Munoz B West SK. The association between myopia and various subtypes of lens opacity: SEE (Salisbury Eye Evaluation) project. Ophthalmology. 2005;112:1395–401. 15953641 10.1016/j.ophtha.2005.02.017

